# Superficial Peritoneal Endometriosis Beyond Surgical Diagnosis: A Narrative Review of Emerging Functional and Molecular Perspectives

**DOI:** 10.3390/medicina62081488

**Published:** 2026-08-02

**Authors:** Mario Palumbo, Giuseppe D’Angelo, Dario Colacurci, Giorgio Maria Baldini, Marco La Verde, Rafał Watrowski, Vito Carone, Giuseppe Bifulco, Pierluigi Giampaolino, Luigi Della Corte

**Affiliations:** 1 Department of Public Health, School of Medicine, University of Naples Federico II, 80138 Naples, Italy; giuseppedangelo1010@gmail.com (G.D.); dariocolacurci@yahoo.it (D.C.);; 2 Department of Biomedical Sciences and Human Oncology, University of Bari “Aldo Moro”, Piazza Giulio Cesare 11, 70124 Bari, Italy; gbaldini97@gmail.com; 3 Obstetrics and Gynecology Unit, Department of Woman, Child and General and Specialized Surgery, University of Campania “Luigi Vanvitelli”, 80138 Naples, Italy; marco.laverde@unicampania.it; 4 Department of Gynecology, Helios Hospital Müllheim, Heliosweg 1, 79379 Müllheim, Germany; 5 Faculty of Medicine, University of Freiburg, 79106 Freiburg, Germany; 6 Gynecologic Oncology Unit, Department of Obstetrics and Gynecology, “F. Miulli” General Regional Hospital, Acquaviva delle Fonti, 70021 Bari, Italy; vito.carone1979@gmail.com; 7 Department of Neuroscience, Reproductive Sciences and Dentistry, School of Medicine, University of Naples “Federico II”, 80131 Naples, Italy; dellacorte.luigi25@gmail.com

**Keywords:** superficial peritoneal endometriosis, diagnostic laparoscopy, dynamic transvaginal ultrasound, neuroinflammation, molecular biomarkers, functional stratification

## Abstract

*Background and Objectives*: Superficial peritoneal endometriosis (SPE) remains one of the most difficult endometriosis phenotypes to diagnose non-invasively, because lesions are frequently small, multifocal, and poorly detectable using conventional imaging. Diagnostic laparoscopy therefore remains the reference standard for direct visualization of superficial peritoneal lesions. However, the inconsistent relationship between visible lesion burden and pain severity, together with the multifactorial nature of chronic pelvic pain, highlights the limitations of a purely lesion-based diagnostic model. This narrative review reassesses SPE from a cautious functional and molecular perspective, focusing on clinically established evidence, emerging but incompletely validated tools, and hypothesis-generating concepts. The novelty of this review lies in its specific focus on SPE as an unresolved diagnostic phenotype and in the proposed integration of surgical diagnosis, expert imaging, pain phenotyping, empirical treatment response, and molecular research within a non-replacement framework. *Materials and Methods*: A narrative literature review was performed using PubMed/MEDLINE, Scopus, Web of Science, and the Cochrane Library for English-language articles published between January 2010 and April 2026. The search focused on SPE, diagnostic laparoscopy, dynamic transvaginal ultrasound, sliding sign, chronic pelvic pain, hormonal treatment response, neuroinflammation, liquid biopsy, circulating biomarkers, epigenetics, and microbiome research. *Results*: Dynamic transvaginal ultrasound, sliding sign assessment, pelvic organ mobility evaluation, and tenderness-guided examination may provide indirect functional information in selected patients with suspected SPE, but they remain operator-dependent and insufficiently standardized for this phenotype. Response to hormonal therapy may support clinical reasoning, but it has low specificity and may also reflect improvement of adenomyosis, primary dysmenorrhea, ovulation-related pain, abnormal uterine bleeding, or other estrogen-sensitive conditions. Liquid biopsy and molecular biomarkers, including circulating microRNAs, extracellular vesicles, cell-free DNA, inflammatory mediators, epigenetic signatures, adipokine-related markers, and microbiome-related signals, remain investigational and require phenotype-specific validation. *Conclusions*: Functional and molecular stratification of suspected SPE represents a promising research direction rather than a current clinical standard. Laparoscopy remains essential when definitive diagnosis or surgical treatment is required, particularly in patients with infertility, refractory symptoms, suspicious imaging, or suspected complex disease. Future validated models may help integrate clinical phenotype, expert imaging, treatment response, pain mechanisms, and molecular profiles to support more individualized and selective diagnostic pathways.

## 1. Introduction

Endometriosis is a chronic estrogen-dependent inflammatory disease characterized by the presence of endometrium-like tissue outside the uterine cavity, affecting approximately 10% of women of reproductive age and representing one of the leading causes of chronic pelvic pain, dysmenorrhea, dyspareunia, infertility, and impaired quality of life [1,2,3,4,5]. Traditionally, the disease has been classified into three major phenotypes: ovarian endometriosis, deep infiltrating endometriosis (DIE), and superficial peritoneal endometriosis (SPE) [6,7,8]. Among these entities, superficial peritoneal endometriosis remains the most difficult to identify, characterize, and manage.

Unlike ovarian endometriomas and DIE, which often present with relatively recognizable imaging findings, SPE is characterized by small peritoneal implants that may appear as transparent, red, white, fibrotic, vesicular, or pigmented lesions scattered across the pelvic peritoneum [9,10,11]. These lesions are frequently multifocal, biologically heterogeneous, and often poorly detectable using conventional imaging modalities. Direct visualization of superficial peritoneal lesions remains the most practical and reliable approach for identifying these lesions. Consequently, diagnostic laparoscopy continues to represent the reference standard for their detection and characterization [12,13,14].

However, increasing evidence suggests that the traditional lesion-based surgical paradigm presents important limitations. In clinical practice, the anatomical extent of superficial lesions frequently correlates poorly with symptom severity. Some women with minimal peritoneal implants experience severe and disabling pelvic pain, whereas others with more extensive disease remain minimally symptomatic or asymptomatic [15,16,17,18]. This discrepancy challenges the assumption that visible lesion burden alone adequately reflects disease activity or explains symptom generation.

Thus, transvaginal ultrasound represents the gold-standard imaging approach for the diagnosis and mapping of ovarian endometriosis and deep infiltrating endometriosis. Nevertheless, the evaluation of superficial peritoneal endometriosis remains a significant diagnostic challenge, largely due to the limited direct visualization of superficial peritoneal lesions [12]. Importantly, this limitation should not be interpreted as evidence that all pelvic pain with negative imaging represents occult SPE. Chronic pelvic pain is multifactorial, and alternative or overlapping conditions such as adenomyosis, pelvic floor dysfunction, irritable bowel syndrome, bladder pain syndrome, vulvodynia, fibromyalgia, pelvic inflammatory disorders, and central sensitization syndromes should be considered early in the diagnostic pathway.

At the same time, the growing recognition of the chronic and multifactorial nature of endometriosis has progressively shifted attention toward the biological and functional mechanisms underlying pain persistence. Neuroinflammation, peripheral and central sensitization, immune dysregulation, hormonal imbalance, angiogenesis, and epigenetic alterations are increasingly recognized as key contributors to symptom generation, even in the presence of minimal or macroscopically subtle disease [19,20,21,22,23,24]. In this context, the pelvic peritoneum should not be interpreted exclusively as a passive anatomical surface, but rather as a dynamic neuroimmunological interface that may contribute to chronic inflammatory and nociceptive signaling.

These considerations are particularly relevant in young women presenting with chronic pelvic pain but negative imaging findings. In such patients, the clinical dilemma remains substantial: whether to proceed with diagnostic laparoscopy despite the absence of detectable endometriomas or DIE, or whether empirical medical management may represent a more appropriate initial strategy. While laparoscopy may identify occult superficial disease in a subset of patients, it is also associated with procedural risks, economic burden, postoperative adhesions, and the possibility of negative or clinically inconclusive findings [25,26,27,28]. Moreover, repeated surgical procedures may not necessarily improve long-term pain outcomes, particularly in patients whose symptoms are driven by persistent neuroinflammatory or sensitization mechanisms rather than by extensive anatomical disease alone [29,30,31].

In parallel, advances in ultrasound technology and functional pelvic assessment have partially modified the traditional diagnostic approach. Although direct sonographic visualization of superficial peritoneal lesions remains limited, dynamic transvaginal ultrasound, sliding sign evaluation, compartmental pelvic mapping, pelvic organ mobility assessment, and tenderness-guided examination may allow the identification of indirect signs of pelvic adhesions, organ fixation, impaired mobility, and site-specific pain responses [32,33,34,35,36]. However, these techniques should be interpreted with caution in suspected SPE, because they are not yet standardized diagnostic tools for superficial lesions and currently lack clearly defined sensitivity, specificity, reproducibility, and validated clinical thresholds for this phenotype.

At the molecular level, growing evidence supports the concept that biological mechanisms may persist beyond visible lesions. Endometriosis is characterized by chronic inflammatory activation, altered immune surveillance, neuroangiogenesis, progesterone resistance, and epigenetic reprogramming capable of sustaining persistent nociceptive activity [37,38,39,40,41,42]. Within this framework, superficial peritoneal endometriosis may be interpreted not merely as an anatomical entity, but also as a condition in which inflammatory, hormonal, immune, and neuroangiogenic mechanisms may contribute to symptoms. Nevertheless, this interpretation remains conceptual and should not be considered a validated clinical diagnostic category.

This evolving perspective has opened increasing interest in the development of non-invasive and biologically oriented diagnostic strategies. Circulating inflammatory mediators, microRNAs, extracellular vesicles, cell-free DNA, microbiome signatures, and epigenetic biomarkers are emerging as potential tools for identifying active disease and stratifying patients according to underlying biological mechanisms rather than surgical staging alone [43,44,45,46,47]. However, these approaches remain investigational. Their clinical interpretation is limited by heterogeneity of endometriosis phenotypes, menstrual cycle phase, BMI, infertility status, hormonal treatment exposure, disease stage, previous surgery, and type of biological material analyzed.

For the purposes of this review, the term “biologically active SPE” is used as a provisional multidimensional construct. It refers to suspected or confirmed superficial peritoneal disease in which symptoms or functional impairment are presumed to be sustained by one or more active biological processes, including local inflammation, neuroangiogenesis, immune dysregulation, estrogen-dependent activity, progesterone resistance, peripheral sensitization, and/or molecular alterations. Pain alone is not sufficient to define biologically active SPE, because chronic pelvic pain may arise from multiple non-endometriotic or overlapping conditions. Similarly, response to hormonal therapy, dynamic ultrasound findings, or circulating biomarkers cannot currently confirm biologically active SPE in isolation.

**Figure 1:**
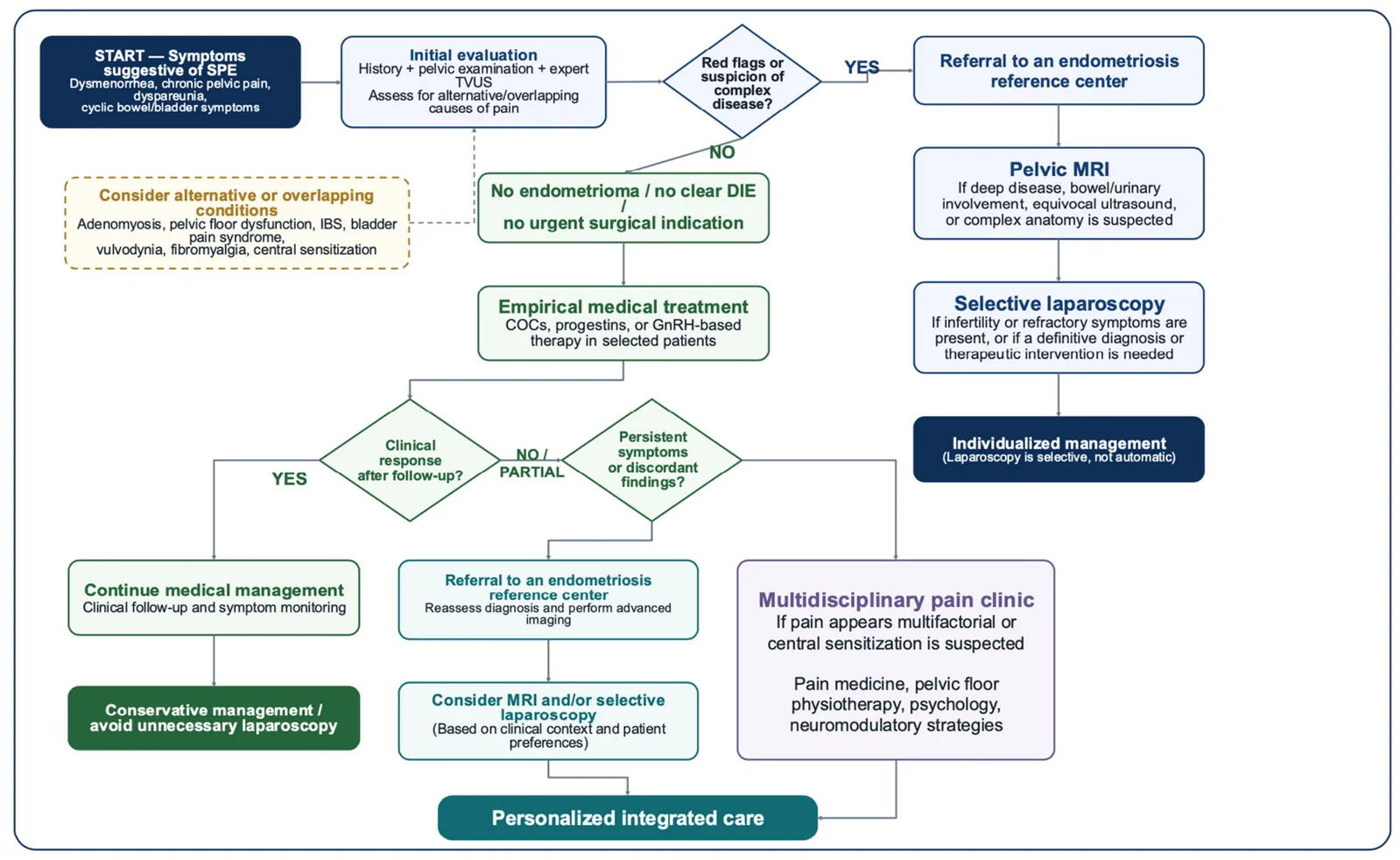
Proposed clinical decision pathway for suspected superficial peritoneal endometriosis (SPE). The figure summarizes an individualized approach to suspected SPE, integrating clinical evaluation, expert TVUS, exclusion of overlapping pain conditions, empirical medical treatment, referral to an endometriosis reference center, MRI, selective laparoscopy, and multidisciplinary pain care when appropriate. The pathway is intended as a conceptual clinical framework and not as a validated diagnostic algorithm. COCs, combined oral contraceptives; DIE, deep infiltrating endometriosis; GnRH, gonadotropin-releasing hormone; IBS, irritable bowel syndrome; MRI, magnetic resonance imaging; SPE, superficial peritoneal endometriosis; TVUS, transvaginal ultrasound.

The challenge, therefore, is not simply to identify visible lesions, but to understand which patients may benefit from surgical confirmation and which may be more appropriately managed through conservative, medical, imaging-based, or multidisciplinary pain-oriented pathways. Within this context, the role of diagnostic laparoscopy should not be abandoned, but rather integrated into a more selective and clinically individualized pathway. Figure 1 summarizes a proposed decision framework for suspected SPE, including empirical treatment, referral to an endometriosis reference center, MRI, selective laparoscopy, and multidisciplinary pain care.

The present narrative review aims to critically reassess superficial peritoneal endometriosis through a cautious functional and molecular perspective. Particular attention is devoted to the limitations of a purely lesion-based surgical paradigm, the potential and limitations of dynamic pelvic ultrasound and functional soft markers, the neuroinflammatory basis of pain generation, the low specificity of response to empirical hormonal therapy, and the emerging role of molecular and liquid biopsy approaches. The novelty of this review lies in its specific focus on SPE as an unresolved diagnostic phenotype and in the proposed integration of clinically established evidence, emerging but incompletely validated functional tools, and hypothesis-generating molecular concepts.

The future challenge may therefore be not simply to diagnose more lesions, but to identify which patients truly require surgical confirmation and which may be managed through a less invasive, biologically informed, and economically sustainable approach [48].

## 2. Materials and Methods

This article was designed as a narrative review with a conceptual and critical synthesis of the available literature on superficial peritoneal endometriosis (SPE), with particular emphasis on diagnostic uncertainty, functional pelvic assessment, chronic pelvic pain, and emerging molecular perspectives. The review was not intended as a systematic review or meta-analysis; therefore, no formal quantitative synthesis, risk-of-bias assessment, or pooled analysis was performed. Nevertheless, the methodological approach was structured to improve transparency, reproducibility, and clarity of literature selection.

A comprehensive literature search was performed using PubMed/MEDLINE, Scopus, Web of Science, and the Cochrane Library. The search included articles published from January 2010 to April 2026. Additional relevant publications were identified through manual screening of the reference lists of selected articles, major reviews, clinical guidelines, and consensus documents.

The search strategy combined Medical Subject Headings, where available, and free-text terms related to superficial peritoneal endometriosis, diagnostic laparoscopy, functional imaging, chronic pelvic pain, biological disease activity, and molecular biomarkers. The following search terms and combinations were used: “superficial peritoneal endometriosis”, “peritoneal endometriosis”, “diagnostic laparoscopy”, “transvaginal ultrasound”, “dynamic transvaginal ultrasound”, “sliding sign”, “pelvic organ mobility”, “tenderness-guided ultrasound”, “soft markers”, “chronic pelvic pain”, “endometriosis-associated pain”, “adenomyosis”, “pelvic floor dysfunction”, “irritable bowel syndrome”, “bladder pain syndrome”, “central sensitization”, “neuroinflammation”, “peripheral sensitization”, “hormonal treatment”, “combined oral contraceptives”, “progestins”, “GnRH agonists”, “GnRH antagonists”, “treatment response”, “liquid biopsy”, “circulating biomarkers”, “microRNA”, “extracellular vesicles”, “exosomes”, “cell-free DNA”, “epigenetics”, “microbiome”, and “multi-omics”. Boolean operators “AND” and “OR” were used to combine terms and adapt the search strategy to each database.

Eligible publications included original clinical studies, observational studies, diagnostic accuracy studies, translational studies, mechanistic studies, clinical guidelines, consensus statements, systematic reviews, and narrative reviews when directly relevant to the diagnosis, biological interpretation, or management of suspected SPE. Articles were considered eligible when they addressed at least one of the following domains: the role and limitations of diagnostic laparoscopy in suspected SPE; the relationship between superficial lesions and pain; dynamic or functional transvaginal ultrasound; sliding sign and pelvic organ mobility; tenderness-guided ultrasound; chronic pelvic pain and overlapping pain syndromes; empirical hormonal treatment and treatment response; neuroinflammatory, immune, hormonal, or epigenetic mechanisms of endometriosis-associated pain; liquid biopsy and circulating biomarkers; microbiome-related mechanisms; or future stratification models.

Articles were excluded if they were not published in English, were conference abstracts without full text, were non-peer-reviewed publications, lacked relevance to SPE or chronic pelvic pain, or focused exclusively on animal or in vitro models without clear translational relevance to human endometriosis. Studies addressing endometriosis in general were included only when their findings were applicable to SPE, chronic pelvic pain mechanisms, non-invasive assessment, or molecular stratification.

The literature selection was performed in two sequential steps. First, titles and abstracts were screened for relevance to the scope of the review. Second, potentially relevant articles were assessed in full text. Particular priority was given to publications addressing clinically relevant diagnostic uncertainty in suspected SPE, limitations of current diagnostic tools, mechanisms of pain generation, non-invasive or minimally invasive assessment, and future patient stratification.

Because this was a narrative review rather than a systematic review, study selection was not intended to be exhaustive in the manner of a PRISMA-based analysis, and a formal flow diagram of record identification and exclusion was not produced. Instead, the aim was to provide a balanced and critical synthesis of the most relevant clinical and translational evidence. To improve transparency, the search strategy, eligibility criteria, exclusion criteria, and thematic organization of the selected literature are reported in detail.

Data were extracted narratively and organized according to predefined thematic areas: diagnostic laparoscopy and its limitations; functional assessment of the pelvic peritoneum; dynamic transvaginal ultrasound and soft markers; neuroinflammatory and molecular mechanisms of SPE-associated pain; empirical hormonal treatment as a low-specificity functional signal; liquid biopsy and circulating biomarkers; microbiome-related mechanisms; and future integrated diagnostic models. Special attention was paid to the distinction between clinically established evidence, emerging but incompletely validated tools, and hypothesis-generating concepts.

Interpretative statements were deliberately framed with cautious wording. Dynamic transvaginal ultrasound, sliding sign assessment, pelvic organ mobility evaluation, tenderness-guided ultrasound, response to hormonal therapy, and molecular biomarkers are discussed as adjunctive or investigational elements that may support future patient stratification, but not as validated substitutes for diagnostic laparoscopy. Diagnostic laparoscopy is considered the reference standard for the direct visualization of superficial peritoneal lesions and remains essential in selected clinical scenarios, including infertility, refractory symptoms, suspicious imaging findings, suspected complex disease, or need for definitive diagnosis and treatment.

## 3. Why a Purely Surgical Paradigm May Be Incomplete

### 3.1. The Historical and Current Role of Diagnostic Laparoscopy in Suspected SPE

For decades, diagnostic laparoscopy has represented a cornerstone in the diagnosis of endometriosis, particularly in patients with suspected superficial peritoneal disease. The ability to directly inspect the pelvic cavity and identify peritoneal implants led to its recognition as the diagnostic reference standard, especially before the widespread development of advanced imaging techniques [48,49,50]. Through laparoscopic exploration, surgeons may identify characteristic superficial lesions, including red flame-like implants, black “powder-burn” lesions, white fibrotic plaques, vesicular or transparent lesions, adhesions, and inflammatory peritoneal defects [9,10,11].

Historically, surgical confirmation was considered essential not only for diagnosis but also for disease classification and therapeutic planning. Histological validation of suspicious lesions further reinforced the central role of laparoscopy within traditional diagnostic algorithms [13,51]. In many patients, surgery allows symptom explanation, anatomical staging, and simultaneous therapeutic intervention through lesion excision or ablation [52,53,54]. Furthermore, diagnostic and therapeutic laparoscopy remains indispensable in selected clinical scenarios, particularly in the presence of infertility, severe or refractory symptoms, bowel or urinary tract involvement, adnexal masses requiring differential diagnosis, suspicious imaging findings, or failure of conservative management [55,56,57].

The purpose of this review is therefore not to challenge the value of laparoscopy. Rather, it is to highlight that a diagnostic approach based exclusively on visible lesion identification may not fully capture the clinical, biological, and functional complexity of superficial peritoneal endometriosis. In this sense, the surgical paradigm remains essential, but may be incomplete when used as the only interpretative framework for symptom generation and patient stratification.

### 3.2. Limitations of a Purely Lesion-Based Diagnostic Model

One of the main limitations of a purely lesion-based model is the inconsistent relationship between anatomical findings and symptom severity. In clinical practice, visible lesion burden frequently correlates poorly with pain intensity, quality-of-life impairment, or long-term symptom persistence [15,16,17,18]. Women with minimal superficial lesions may experience severe dysmenorrhea, chronic pelvic pain, or deep dyspareunia, whereas more extensive disease may occasionally remain minimally symptomatic or asymptomatic.

This discrepancy suggests that visible lesions alone may not fully explain pain generation or disease activity. Chronic inflammation, neuroangiogenesis, peripheral sensitization, central nervous system modulation, immune dysfunction, and hormonal signaling may contribute substantially to symptom persistence [19,20,21,22,23,24]. Consequently, laparoscopy may identify anatomical manifestations of disease, but it cannot directly measure the biological activity of lesions or the broader neuroinflammatory mechanisms that may sustain pain.

Another important limitation concerns the intrinsic heterogeneity of superficial lesions themselves. Superficial peritoneal implants may differ substantially in vascularization, inflammatory activity, hormonal responsiveness, fibrosis, innervation density, and molecular profile [58,59,60]. Some lesions may represent active inflammatory foci capable of generating nociceptive signaling despite their small size, while others may reflect relatively inactive or fibrotic residual disease. Standard laparoscopic morphology alone cannot reliably distinguish these biological states.

Moreover, laparoscopic visualization itself remains imperfect. Minimal lesions may be subtle, transparent, multifocal, or located in anatomically difficult areas, potentially contributing to underdiagnosis or interobserver variability [61]. Conversely, the identification of small superficial implants does not necessarily establish a direct causal relationship between those lesions and the patient’s symptoms. This issue is particularly relevant in women with chronic overlapping pain disorders, pelvic floor dysfunction, irritable bowel syndrome, bladder pain syndrome, adenomyosis, or central sensitization syndromes [62,63,64]. Therefore, surgical findings should always be interpreted within a broader clinical and pain-phenotype context.

### 3.3. Young Women with Pelvic Pain and Negative Imaging

The limitations of a purely lesion-based paradigm become particularly evident in young women presenting with chronic pelvic pain but negative imaging findings. This clinical scenario is increasingly common in gynecological practice and represents one of the most challenging areas of endometriosis management.

Many of these patients report severe dysmenorrhea, cyclic bowel symptoms, dyspareunia, chronic pelvic discomfort, or functional impairment despite the absence of ovarian endometriomas or deep infiltrating lesions on transvaginal ultrasound or magnetic resonance imaging [65,66,67]. In such cases, superficial peritoneal endometriosis may be clinically suspected, yet its confirmation remains difficult because of the limited sensitivity of imaging modalities for minimal peritoneal disease [32,33,34,35,36].

Traditionally, these patients were often referred for diagnostic laparoscopy to exclude occult endometriosis. However, this strategy requires careful individualization. A proportion of laparoscopies may reveal either minimal superficial lesions of uncertain clinical significance or entirely negative findings [68,69,70]. Moreover, even when superficial lesions are identified and surgically treated, symptom persistence or recurrence remains relatively common, suggesting that pain mechanisms may extend beyond visible disease alone [29,30,31].

This issue is particularly relevant in adolescents and young reproductive-age women, in whom repeated surgical interventions may expose patients to procedural risks, postoperative adhesions, ovarian reserve concerns, healthcare costs, and psychological burden [71,72,73]. At the same time, delaying symptom-directed treatment while awaiting surgical confirmation may contribute to prolonged pain chronification and reduced quality of life. These considerations support a more selective diagnostic strategy that integrates clinical phenotype, expert imaging, empirical medical treatment, and multidisciplinary pain assessment when appropriate.

### 3.4. The Risk of Unnecessary or Non-Informative Laparoscopy

Although laparoscopy is generally considered safe when performed by experienced surgeons, it remains an invasive surgical procedure associated with non-negligible risks. Complications such as bleeding, infection, vascular injury, bowel or urinary tract damage, postoperative adhesions, anesthesia-related morbidity, and chronic postoperative pain may occur even in experienced hands [74,75,76]. While major complications remain relatively uncommon, their potential impact is particularly relevant in young patients undergoing surgery primarily for diagnostic purposes.

Beyond procedural risks, unnecessary or non-informative laparoscopies may also contribute to substantial healthcare burden. Endometriosis already represents a major socioeconomic challenge due to diagnostic delay, repeated consultations, chronic pain management, fertility treatments, and reduced productivity [77,78,79]. Performing surgery in patients who may instead benefit from medical management, conservative follow-up, or multidisciplinary pain care may further increase costs without necessarily improving long-term outcomes.

An additional concern relates to the psychological implications of surgery. Negative laparoscopic findings may generate frustration, perceived invalidation of symptoms, or diagnostic uncertainty for patients experiencing chronic pain [80]. Conversely, the identification of minimal superficial lesions does not always clarify whether the observed abnormalities fully explain symptom severity, potentially leading to persistent uncertainty even after surgery.

Repeated laparoscopic procedures may also contribute to disease complexity through postoperative adhesions, altered pelvic anatomy, and persistent nociceptive sensitization [81]. These considerations do not reduce the value of laparoscopy when clinically indicated, but they reinforce the concept that diagnostic surgery should not be regarded as an automatic endpoint in all women with suspected superficial peritoneal disease.

### 3.5. Biological Activity Beyond Visible Lesions: A Provisional Concept

Growing evidence suggests that symptoms in superficial peritoneal endometriosis may be influenced not only by the presence of visible lesions but also by inflammatory, neuroangiogenic, immune, hormonal, and sensitization mechanisms. Peritoneal fluid in affected women demonstrates increased concentrations of inflammatory cytokines, prostaglandins, growth factors, oxidative stress mediators, and neuroangiogenic factors [82,83,84,85]. These molecules may contribute to peripheral nerve sensitization, immune cell recruitment, vascular proliferation, and chronic inflammatory signaling.

At the same time, increased density of sensory nerve fibers and neurotrophic mediators within superficial lesions may amplify nociceptive responses even in the presence of limited anatomical disease [86,87,88]. Central sensitization mechanisms may further perpetuate chronic pain independently of active lesion progression. Persistent nociceptive input originating from inflammatory pelvic pathways may induce long-term alterations in central nervous system processing, resulting in pain amplification, hyperalgesia, and symptom persistence despite limited or surgically treated disease [89,90,91].

Within this framework, the term “biologically active superficial peritoneal endometriosis” may be useful as a provisional conceptual construct. In this review, it refers to suspected or confirmed superficial disease in which symptoms or functional impairment are presumed to be sustained by one or more active biological processes, including local inflammation, neuroangiogenesis, immune dysregulation, estrogen-dependent activity, progesterone resistance, peripheral sensitization, and possibly molecular alterations.

However, this concept should be interpreted with caution. Pain alone is not sufficient to define biologically active SPE, because chronic pelvic pain may result from multiple non-endometriotic or overlapping conditions. Similarly, response to hormonal treatment, dynamic ultrasound findings, or circulating biomarkers cannot currently confirm biologically active SPE in isolation. At present, biologically active SPE should be regarded as a hypothesis-generating framework requiring future validation rather than a clinically established diagnostic category.

### 3.6. Toward a More Selective and Integrated Diagnostic Framework

Taken together, these observations support the need for a more selective and integrated diagnostic framework rather than replacement of surgical diagnosis. Within this evolving perspective, diagnosis should not rely exclusively on the visualization of macroscopic lesions, but may benefit from the integration of multiple dimensions, including symptom phenotype, dynamic pelvic assessment, indirect functional markers, hormonal responsiveness, inflammatory mechanisms, and emerging molecular signatures [92,93,94,95].

This conceptual shift does not imply abandoning surgery. Diagnostic and therapeutic laparoscopy remain fundamental tools in carefully selected patients, particularly in the presence of infertility, severe refractory symptoms, suspicion of deep disease, organ involvement, suspicious imaging findings, or failure of empirical treatment [55,56,57]. However, it suggests that surgical exploration should be more selective and clinically individualized rather than universally applied.

The future challenge is therefore not simply to identify every superficial implant, but to distinguish patients who are most likely to benefit from surgical confirmation and treatment from those in whom surgery may provide limited benefit. Within this framework, functional ultrasound evaluation, response to medical therapy, and molecular stratification may eventually contribute to a more personalized and less invasive diagnostic pathway, but only after adequate validation.

## 4. Functional Evaluation of the Pelvic Peritoneum: Potential and Limitations

### 4.1. The Pelvic Peritoneum as a Dynamic Functional Interface

The pelvic peritoneum has traditionally been regarded primarily as an anatomical surface onto which endometriotic implants adhere and proliferate. However, growing evidence suggests that the peritoneal environment may also play an active role in the pathophysiology of superficial peritoneal endometriosis. The pelvic peritoneum represents a dynamic biological interface in which immune cells, inflammatory mediators, sensory nerve fibers, vascular structures, hormonal signaling, and extracellular matrix components interact [96,97,98,99].

Within this environment, even minimal superficial lesions may be associated with inflammatory and neuroangiogenic activation. Increased concentrations of cytokines, prostaglandins, growth factors, macrophages, mast cells, and oxidative stress mediators within peritoneal fluid may contribute to chronic nociceptive activation and altered pelvic organ function [82,83,84,85]. These mechanisms provide a biological rationale for evaluating not only visible lesions but also pelvic function, mobility, and site-specific pain responses.

In parallel, chronic inflammation may influence the biomechanical properties of pelvic structures. Repeated inflammatory activation can promote fibrosis, adhesions, altered tissue compliance, organ fixation, and impaired mobility of pelvic compartments [100,101,102]. Consequently, the pelvic cavity may be interpreted not only as a static anatomical space but also as a functional system whose dynamic behavior may provide indirect information regarding underlying pelvic pathology.

This interpretation, however, requires caution. Functional abnormalities of the pelvic peritoneum are not specific to SPE. Reduced mobility, adhesions, focal tenderness, or compartmental stiffness may also occur after previous surgery, pelvic inflammatory disease, adenomyosis, non-endometriotic adhesions, pelvic floor dysfunction, irritable bowel syndrome, or other chronic pelvic pain conditions. Therefore, functional pelvic assessment should be considered an adjunctive component of clinical evaluation rather than an independent diagnostic test for superficial peritoneal lesions.

### 4.2. Dynamic Transvaginal Ultrasound

Over the last decade, transvaginal ultrasound has evolved from a predominantly morphological technique into a more dynamic and function-oriented examination [32,33,34,35,36]. Conventional ultrasound remains highly effective for the detection and mapping of ovarian endometriomas and several forms of deep infiltrating endometriosis. In contrast, its sensitivity for minimal superficial peritoneal lesions remains limited because these implants are often small, flat, subtle, multifocal, or located on peritoneal surfaces that are difficult to assess sonographically [103,104,105].

Dynamic transvaginal ultrasound has partially expanded the role of pelvic imaging by allowing real-time assessment of pelvic organ mobility, adhesions, compartment flexibility, and pain responses. Through gentle manipulation of the transvaginal probe and, when appropriate, external abdominal pressure, the examiner may evaluate the reciprocal gliding of pelvic structures and identify abnormalities suggestive of altered pelvic mechanics [106,107,108].

This dynamic approach shifts the focus from direct lesion visualization toward the assessment of functional pelvic behavior. In selected patients with suspected SPE and negative conventional imaging, abnormalities may manifest not as clearly identifiable nodules or cysts, but as subtle reductions in tissue mobility, localized tenderness, altered compartment interaction, or restricted organ sliding [109].

Nevertheless, dynamic transvaginal ultrasound should not be presented as a validated diagnostic model for SPE. At present, its role in superficial peritoneal disease remains incompletely standardized, and robust data defining sensitivity, specificity, interobserver reproducibility, and clinically actionable thresholds are still lacking. Therefore, dynamic ultrasound findings should be interpreted as indirect functional observations that may support clinical reasoning, guide referral, or contribute to individualized decision-making, but not replace laparoscopy when definitive diagnosis or treatment is required.

### 4.3. Sliding Sign and Pelvic Organ Mobility

Among dynamic ultrasound techniques, evaluation of the sliding sign has emerged as one of the most clinically relevant indirect markers of pelvic adhesions and posterior compartment involvement [110,111,112]. The sliding sign assesses the ability of pelvic organs to glide freely against one another during probe manipulation or external pressure. Under physiological conditions, structures such as the uterus, rectum, sigmoid colon, ovaries, and posterior vaginal fornix demonstrate preserved mobility within the pelvic cavity.

A negative or reduced sliding sign indicates impaired mobility and possible adhesion formation, often reflecting inflammatory changes involving the pouch of Douglas or adjacent peritoneal surfaces [113,114,115,116]. Although sliding sign assessment has been more extensively studied in the context of deep infiltrating endometriosis and pouch of Douglas obliteration, it may also provide indirect information in patients with suspected superficial disease when subtle adhesions or localized inflammatory reactions alter normal pelvic organ dynamics.

Abnormalities of organ mobility may sometimes precede overt structural distortion visible on conventional imaging. Minimal inflammatory adhesions, focal fibrosis, or subtle peritoneal involvement may interfere with tissue gliding even in the absence of large nodules or endometriomas [105,117,118,119,120]. This concept is clinically relevant in women with severe symptoms but apparently normal pelvic anatomy on static ultrasound examination.

Ovarian mobility assessment represents another component of functional pelvic evaluation. Reduced ovarian sliding, partial fixation to the pelvic sidewall, limited mobility relative to the uterus, or altered response to gentle probe pressure may suggest adhesive disease involving the ovarian fossa or posterior compartment [21,22,23,33]. However, these findings are not specific to SPE and may overlap with previous surgery, pelvic inflammatory disease, non-endometriotic adhesions, adenomyosis, or anatomical variation.

Therefore, sliding sign and organ mobility assessment should not be interpreted as diagnostic proof of superficial endometriosis. Their main clinical value lies in providing indirect information regarding pelvic mechanics and possible adhesions, which should be integrated with symptoms, clinical history, expert imaging findings, fertility goals, response to treatment, and the presence or absence of alternative pain generators.

### 4.4. Tenderness-Guided Ultrasound

Tenderness-guided transvaginal ultrasound integrates anatomical imaging with real-time symptom provocation, allowing the examiner to correlate site-specific pain responses with pelvic anatomical structures [23]. During ultrasound examination, targeted pressure applied with the transvaginal probe may reproduce the patient’s symptoms in specific pelvic compartments, particularly within the posterior fornix, uterosacral ligament region, ovarian fossae, or pouch of Douglas.

The elicitation of focal tenderness in these areas may increase clinical suspicion of occult inflammatory, adhesive, or neurogenic pelvic disease when overt structural abnormalities are absent [29,30,31,32]. This approach may be particularly informative in suspected superficial endometriosis, where visible lesions may be minimal or undetectable but inflammatory or sensitization mechanisms may contribute to pain generation [16,42].

However, pain provocation during ultrasound is intrinsically non-specific. It may reflect local inflammatory activity, peripheral sensitization, pelvic floor hypertonicity, visceral hypersensitivity, central sensitization, anxiety-related pain amplification, or other overlapping pain syndromes. Increased sensory nerve fiber density, mast cell activation, local cytokine production, and peripheral sensitization may amplify nociceptive responses to mechanical stimulation [99,100], but these mechanisms are not unique to SPE.

Consequently, tenderness-guided ultrasound should be considered a functional extension of the clinical pelvic examination rather than a stand-alone diagnostic test. Its findings may help localize pain, guide further evaluation, and support multidisciplinary decision-making, but they should not be used in isolation to confirm superficial peritoneal endometriosis.

### 4.5. Functional Soft Markers of Suspected Occult Peritoneal Disease

The growing use of dynamic and tenderness-guided ultrasound has led to the description of multiple indirect “soft markers” suggestive of possible occult peritoneal or adhesive disease [80,81]. Unlike classic imaging signs such as endometriomas or deep nodules, these markers do not directly visualize superficial lesions. Instead, they reflect functional alterations of pelvic structures potentially associated with chronic inflammatory or adhesive processes [42].

Commonly described soft markers include reduced sliding sign, ovarian fixation, compartment stiffness, altered uterine mobility, focal tenderness, site-specific pain elicitation, asymmetric organ movement, subtle adhesions involving the posterior compartment or ovarian fossae, reduced bowel sliding, and mild ovarian medialization [45,46,47,48]. Individually, these findings have limited specificity. Their combined presence may increase clinical suspicion, but they cannot currently establish a diagnosis of SPE.

The interpretation of functional soft markers should therefore be integrated within a broader clinical context that includes symptom phenotype, menstrual pattern, pain distribution, gastrointestinal or urinary symptoms, quality-of-life impairment, previous surgery, infertility status, and response to empirical treatment [47,65,66,67]. In this regard, functional ultrasound may contribute to “dynamic pelvic phenotyping”, but this concept remains exploratory and requires prospective validation.

Several limitations must be emphasized. Dynamic ultrasound assessment is highly operator-dependent, lacks complete standardization for superficial disease, and may demonstrate interobserver variability [73,74,75,76,77]. Furthermore, the same soft markers may be observed in non-endometriotic conditions, including adhesions unrelated to endometriosis, pelvic inflammatory disease, irritable bowel syndrome, pelvic floor dysfunction, adenomyosis, or postsurgical pelvic remodeling.

These limitations do not eliminate the potential clinical value of functional pelvic assessment, but they define its appropriate use. Functional soft markers should be considered adjunctive observations that may help guide clinical reasoning, referral to expert centers, MRI evaluation, empirical treatment, or selective laparoscopy, rather than validated diagnostic criteria for SPE.

### 4.6. Integrating Functional Ultrasound into Clinical Decision-Making

Taken together, dynamic transvaginal ultrasound, sliding sign evaluation, pelvic organ mobility assessment, tenderness-guided examination, and functional soft markers support a broader interpretation of pelvic imaging in suspected superficial endometriosis.

Within this framework, ultrasound should not be viewed exclusively as a tool for identifying visible lesions. In expert hands, it may also provide indirect information regarding pelvic mobility, adhesions, compartmental restriction, site-specific pain, and possible functional consequences of occult peritoneal disease [83,84]. However, this information should always be interpreted in combination with clinical history, symptom phenotype, fertility goals, exclusion of alternative pain generators, and shared decision-making.

Functional ultrasound findings should not be used as substitutes for laparoscopy. Rather, they may contribute to a more selective strategy in which invasive procedures are considered when symptoms persist, infertility is present, imaging is suspicious or discordant, empirical treatment fails, or definitive diagnosis and treatment are required [85,86,87,88].

The future role of ultrasound in SPE may therefore extend beyond morphology toward a more comprehensive assessment of pelvic function. However, before functional ultrasound can be incorporated into validated diagnostic pathways for superficial disease, prospective studies are needed to define standardized protocols, reproducibility, diagnostic performance, thresholds for clinical action, and added value compared with conventional assessment (Table 1).

## 5. Neuroinflammatory and Molecular Mechanisms in SPE-Associated Pain

### 5.1. Local Inflammation and the Peritoneal Microenvironment

Chronic inflammation represents one of the central mechanisms potentially involved in pain generation in superficial peritoneal endometriosis. Although superficial lesions are often anatomically limited, they may coexist with a biologically active inflammatory microenvironment capable of sustaining nociceptive signaling disproportionate to lesion size [100,101,102,103,104]. This observation may help explain the frequently poor correlation between visible superficial lesion burden and symptom severity.

Peritoneal fluid from women with endometriosis has been reported to contain increased concentrations of pro-inflammatory cytokines and chemokines, including interleukin-1β, interleukin-6, interleukin-8, tumor necrosis factor-α, prostaglandins, macrophage migration inhibitory factor, and several growth factors [82,83,84,85]. These mediators may contribute to immune cell recruitment, angiogenesis, oxidative stress, and activation of peripheral nociceptive pathways [105].

At the molecular level, persistent activation of inflammatory transcriptional pathways, particularly nuclear factor kappa B, may amplify inflammatory signaling and support lesion survival [106,107,108,109]. Nuclear factor kappa B activation promotes the expression of cytokines, adhesion molecules, cyclooxygenase-2, prostaglandins, and anti-apoptotic mediators, thereby reinforcing a self-sustaining inflammatory environment.

Inflammatory mediators may also directly influence nociceptive nerve fibers. Cytokines and prostaglandins can reduce activation thresholds of peripheral sensory neurons, resulting in hyperalgesia and amplified pain perception [92]. Repeated inflammatory stimulation may progressively sensitize peripheral nerve endings, contributing to the transition from cyclical menstrual pain to more persistent chronic pelvic pain.

Oxidative stress may further contribute to this process. Increased reactive oxygen species and impaired antioxidant defenses have been described in endometriosis and may promote DNA damage, mitochondrial dysfunction, inflammatory amplification, tissue remodeling, and neuronal sensitization [110].

In the context of SPE, these mechanisms support the biological plausibility that small or subtle superficial lesions may contribute to pain. However, inflammatory activation is not specific to SPE and may also occur in other endometriosis phenotypes, adenomyosis, pelvic inflammatory disorders, postsurgical adhesions, and non-gynecological chronic pelvic pain conditions. Therefore, inflammation should be interpreted as a possible contributor to symptom generation rather than as diagnostic evidence of biologically active SPE.

### 5.2. Neuroangiogenesis and Nerve Fiber Proliferation

One of the most relevant advances in the understanding of endometriosis-associated pain has been the recognition of neuroangiogenesis as a key biological process [115]. Superficial peritoneal lesions may be characterized by increased vascularization and abnormal proliferation of sensory nerve fibers, creating a highly innervated inflammatory microenvironment [105,106,107,108]. This mechanism is particularly relevant for SPE because small lesions may generate significant pain when they are associated with neurotrophic and inflammatory activation [99,100].

Increased expression of vascular endothelial growth factor, nerve growth factor, brain-derived neurotrophic factor, and other neuroangiogenic mediators has been reported within endometriotic lesions and peritoneal fluid [97]. These molecules may promote both neovascularization and the growth of sensory and autonomic nerve fibers into ectopic tissue.

Histological studies have shown that superficial lesions may contain increased densities of small unmyelinated sensory nerve fibers, often in close spatial relationship with inflammatory cells, particularly macrophages and mast cells [105]. This neuroimmune interaction may contribute to persistent nociceptive activation and amplification of pain signaling [111].

Neuroangiogenesis may also be hormonally modulated. Estrogen can stimulate angiogenic and neurogenic pathways, while progesterone resistance may impair anti-inflammatory control mechanisms [106,107,108,109]. Consequently, hormonally active superficial lesions may maintain a neurovascular inflammatory niche responsive to cyclical endocrine fluctuations [121].

These observations provide a biological rationale for site-specific tenderness and pain reproduction during pelvic examination or tenderness-guided ultrasound. Nevertheless, neuroangiogenesis is not unique to SPE and cannot be assessed reliably in routine clinical practice without tissue or advanced translational analyses. It should therefore be discussed as a mechanistic explanation for pain heterogeneity, not as a clinically validated diagnostic marker.

### 5.3. Peripheral and Central Sensitization

Pain generation in superficial peritoneal endometriosis may extend beyond local inflammation alone. Persistent nociceptive input originating from inflammatory peritoneal lesions may progressively induce sensitization of both peripheral and central nervous system pathways [98,99,100].

Peripheral sensitization occurs when repeated inflammatory stimulation lowers the activation threshold of nociceptive afferent fibers. Cytokines, prostaglandins, nerve growth factor, and other inflammatory mediators may alter ion channel activity and neuronal excitability, resulting in exaggerated pain responses to mechanical, menstrual, or visceral stimuli [120]. Clinically, this may manifest as severe dysmenorrhea, deep dyspareunia, bowel discomfort, or diffuse pelvic tenderness despite limited visible disease.

Over time, sustained peripheral nociceptive signaling may contribute to central sensitization. This process involves functional and structural changes within spinal and supraspinal pain-processing pathways, leading to amplification and persistence of pain even when peripheral lesion burden is limited or has been treated [120,121]. Increased excitability of dorsal horn neurons, altered descending inhibitory pathways, and enhanced cortical pain processing may contribute to pain chronification.

Central sensitization may help explain why some patients continue to experience severe pain after apparently adequate surgical treatment or in the absence of extensive anatomical disease [122,123,124,125,126]. In these patients, pain may become partially autonomous from active lesion burden and maintained by persistent neuroimmune and central nervous system alterations [127,128].

This concept has major clinical implications for suspected SPE. Severe pain with negative imaging should not automatically be interpreted as occult superficial disease. It may reflect SPE in some patients, but it may also arise from pelvic floor dysfunction, visceral hypersensitivity, bladder pain syndrome, irritable bowel syndrome, vulvodynia, fibromyalgia, psychological distress, or other overlapping pain mechanisms. Recognition of sensitization should therefore encourage broader pain phenotyping and multidisciplinary assessment, rather than repeated surgery alone.

### 5.4. Immune Dysregulation and Mast Cell Activation

In addition to inflammatory cytokine production, immune dysfunction may contribute to lesion persistence and pain in endometriosis [48]. The peritoneal environment in affected women may be characterized by altered immune surveillance, chronic immune activation, and dysregulated interactions between inflammatory cells and sensory nerve fibers.

Macrophages represent one of the most abundant immune cell populations within the peritoneal cavity in endometriosis. These cells may exhibit a pro-inflammatory and pro-angiogenic phenotype characterized by increased secretion of cytokines, vascular endothelial growth factor, prostaglandins, and neurotrophic mediators [105,106]. Rather than effectively clearing ectopic endometrial cells, activated macrophages may support lesion survival, tissue remodeling, fibrosis, and nociceptive amplification.

Mast cells have also emerged as relevant mediators of pain generation. Increased mast cell density has been identified in superficial lesions and surrounding peritoneal tissue [111,112,113,114,115]. Through the release of histamine, tryptase, prostaglandins, cytokines, and nerve growth factors, mast cells may interact directly with peripheral nerve endings and amplify neuroinflammatory signaling.

This mast cell–nerve interaction may be particularly relevant in patients with severe pain despite minimal lesion burden. Activated mast cells may contribute to peripheral sensitization, neurogenic inflammation, and hyperalgesia even in the absence of extensive anatomical distortion [100].

Natural killer cell dysfunction may further contribute to disease persistence. Reduced natural killer cell cytotoxic activity can impair the clearance of ectopic endometrial cells, allowing continued lesion survival and inflammatory activation [121]. Altered T-cell responses and increased regulatory T-cell activity may also contribute to immune tolerance within the peritoneal environment.

However, immune dysregulation is a broad mechanism and should not be presented as specific to superficial lesions. Similar immune alterations may be observed in other endometriosis phenotypes and in several inflammatory or pain-related disorders. For this reason, immune mechanisms should be interpreted as part of a broader biological framework rather than as SPE-specific diagnostic evidence.

### 5.5. Hormonal and Epigenetic Regulation

Hormonal dysregulation represents another important component in the pathophysiology of endometriosis-associated pain. Endometriotic lesions are characterized by an altered hormonal microenvironment marked by enhanced estrogen responsiveness and impaired progesterone signaling, creating a pro-inflammatory and pro-survival state that may contribute to lesion activity and chronic pain generation [115].

At the molecular level, increased estrogen receptor beta expression, aberrant aromatase activity, and dysregulated local estrogen metabolism may promote persistent estrogenic stimulation within ectopic tissues [115]. Beyond its proliferative effects, estrogen may contribute to inflammation by stimulating cytokine production, angiogenesis, neurogenesis, immune activation, and nociceptive signaling [99].

Conversely, progesterone signaling appears impaired in endometriosis. Reduced progesterone receptor expression and altered downstream progesterone responsiveness may limit the anti-inflammatory, differentiative, and anti-proliferative effects normally exerted by progesterone within endometrial tissue [109]. This condition, commonly referred to as progesterone resistance, may contribute to defective suppression of inflammatory pathways and persistence of biologically active disease.

These hormonal abnormalities may be maintained through epigenetic mechanisms, including DNA methylation, histone modifications, and dysregulated non-coding RNA expression [33,34,35,36]. Epigenetic regulation may stabilize aberrant transcriptional programs involved in inflammation, neuroangiogenesis, immune escape, steroid signaling, and cellular survival. In this context, epigenetic reprogramming may generate a form of persistent molecular memory, allowing inflammatory and nociceptive pathways to remain active over time [129].

In SPE, hormonal and epigenetic mechanisms provide a plausible explanation for clinical heterogeneity, including differences in pain severity, recurrence, and response to treatment. However, epigenetic profiling is not currently available for routine clinical classification of superficial disease. These mechanisms should therefore be presented as part of the future research landscape rather than as clinically actionable markers.

### 5.6. Persistent Pain Beyond Visible Disease

Taken together, these findings support the concept that pain in superficial peritoneal endometriosis cannot be interpreted exclusively as the direct consequence of visible lesion burden. Rather than depending solely on anatomical extent, symptom generation may arise from a complex interaction between chronic inflammation, neuroangiogenesis, immune dysregulation, hormonal imbalance, epigenetic alterations, peripheral sensitization, and central nervous system modulation [93,94,95,96].

This perspective may help explain why some women experience severe and disabling pain despite limited superficial disease at surgery, while others continue to report persistent symptoms even after apparently complete lesion treatment. It may also provide a biological explanation for patients with strong clinical suspicion of endometriosis but negative imaging findings, as well as for recurrence or persistence of pain in the absence of clear anatomical progression.

At the same time, this interpretation must remain cautious. Although laparoscopy remains fundamental for the identification and treatment of visible lesions, it cannot fully capture the biological complexity of chronic pelvic pain or directly evaluate the molecular and neuroinflammatory pathways sustaining symptom persistence [24,25,26,27]. Conversely, the presence of pain, sensitization, or inflammatory features does not prove that SPE is the primary driver of symptoms.

Within this evolving framework, superficial endometriosis may be viewed not only as a structural gynecological condition, but also as a potential chronic inflammatory and neuroimmune phenotype involving dynamic interactions between pelvic tissues, immune cells, hormonal signaling, and pain-processing pathways [129]. Understanding these mechanisms may improve clinical interpretation and future patient stratification, but they do not currently provide a validated diagnostic alternative to laparoscopy.

Future management strategies may eventually combine anatomical, functional, inflammatory, neuroimmune, and molecular assessment to support more personalized decision-making [130,131,132]. Until such approaches are validated, neuroinflammatory and molecular mechanisms should be considered explanatory and hypothesis-generating, helping clinicians interpret symptom heterogeneity while preserving the central role of established diagnostic and therapeutic pathways (Table 2).

## 6. Medical Therapy Response as a Low-Specificity Functional Signal

### 6.1. Empirical Hormonal Therapy in Suspected Superficial Peritoneal Endometriosis

Over recent years, increasing emphasis has been placed on symptom-oriented management in women with suspected endometriosis, particularly in the absence of overt imaging findings. Current international recommendations support the initiation of empirical hormonal therapy in selected symptomatic patients without mandatory surgical confirmation, especially when clinical suspicion is high and no alternative pathology requiring immediate intervention is evident [133].

This approach is particularly relevant for superficial peritoneal endometriosis, where direct visualization of lesions remains difficult both radiologically and surgically. In many young women presenting with dysmenorrhea, chronic pelvic pain, dyspareunia, cyclic gastrointestinal symptoms, or acyclic pelvic discomfort, imaging may fail to demonstrate ovarian endometriomas or deep infiltrating lesions despite clinically significant symptoms [134].

Combined oral contraceptives, continuous progestins, dienogest, levonorgestrel-releasing intrauterine systems, and gonadotropin-releasing hormone agonists or antagonists remain among the main pharmacological options for symptom control in endometriosis-associated pain [134,135]. These treatments primarily act through suppression of ovulation, reduction in estrogen-dependent stimulation, inhibition of inflammatory pathways, and modulation of prostaglandin production.

Hormonal suppression may influence several biological mechanisms involved in pain generation. Reduced estrogenic stimulation may attenuate neuroangiogenesis, inflammatory cytokine production, mast cell activation, prostaglandin synthesis, oxidative stress, and peripheral nociceptive sensitization [135,136]. Therefore, clinical improvement during hormonal treatment may reflect suppression of estrogen-dependent inflammatory pathways rather than reduction in visible lesion burden alone [136].

However, empirical hormonal therapy should be interpreted first as a therapeutic strategy, not as a diagnostic test. Its use may help control symptoms and guide clinical management, but response to treatment cannot confirm the presence of superficial peritoneal endometriosis.

### 6.2. Clinical Responders: Functional Phenotype Rather than Diagnostic Category

Some patients with suspected superficial peritoneal endometriosis experience marked improvement during empirical hormonal therapy despite the absence of surgically confirmed disease [134]. In clinical practice, hormonal treatment may improve dysmenorrhea, chronic non-menstrual pelvic pain, dyspareunia, bowel symptoms, and quality-of-life impairment [133,134,135,136]. From a biological perspective, this response may reflect suppression of estrogen-dependent inflammatory, neuroangiogenic, and nociceptive pathways that contribute to pelvic pain generation [137].

For this reason, clinical response may be considered a functional observation. It may suggest that symptoms are at least partly hormonally modulated and potentially linked to estrogen-dependent inflammatory activity. Nevertheless, this interpretation must remain cautious. A clinical responder should not be automatically classified as having biologically active SPE.

The specificity of treatment response is intrinsically low. Improvement after combined oral contraceptives, progestins, dienogest, or GnRH-based therapies may result from suppression of endometriosis-related inflammation, but it may also reflect improvement of adenomyosis, primary dysmenorrhea, ovulation-related pain, abnormal uterine bleeding, prostaglandin-mediated pain, or other estrogen-sensitive gynecological conditions. In addition, symptom improvement may partly derive from menstrual suppression, reduced bleeding, anti-ovulatory effects, or nonspecific reduction in cyclic pelvic inflammation.

Therefore, response to empirical hormonal therapy should be interpreted as evidence of hormonally modulated pelvic pain rather than diagnostic confirmation of superficial peritoneal endometriosis. Its value lies in supporting individualized clinical reasoning, not in replacing imaging, comprehensive evaluation, or laparoscopy when definitive diagnosis or treatment is required.

### 6.3. Biological Interpretation of Hormonal Response

The biological plausibility of treatment response in suspected SPE is supported by the role of inflammatory and prostaglandin-related pathways in endometriosis-associated pain. Altered prostaglandin signaling and inflammatory mediator expression may contribute to lesion activity, pelvic inflammation, and nociceptive sensitization [138]. Hormonal suppression may attenuate these pathways and thereby reduce pain even when superficial lesions are small, subtle, or not directly visible on imaging.

Within this framework, patients who respond well to hormonal therapy may represent a clinical phenotype characterized by predominantly hormonally responsive inflammatory pathways, limited structural distortion, and relatively reversible peripheral nociceptive activation. This phenotype may be particularly relevant in young women with negative imaging and symptoms suggestive of endometriosis.

However, this remains a hypothesis-generating interpretation. At present, no validated clinical threshold defines a “positive” hormonal response as evidence of SPE. The magnitude, timing, and durability of improvement are not standardized, and placebo effects, natural symptom fluctuation, adherence, analgesic use, and coexisting pelvic pain disorders may influence perceived treatment response.

Thus, hormonal response may contribute to functional phenotyping, but only when interpreted together with symptom pattern, imaging findings, menstrual history, fertility goals, exclusion of alternative diagnoses, and patient preferences.

### 6.4. Partial or Absent Response to Medical Therapy

Partial or absent response to empirical hormonal therapy should not be interpreted simplistically. It does not exclude endometriosis, nor does it automatically indicate the need for immediate diagnostic laparoscopy. Several explanations may account for persistent symptoms despite adequate hormonal suppression.

First, some patients may have surgically relevant endometriosis or adhesions that are not adequately controlled by medical therapy. In selected cases, surgical treatment remains appropriate, particularly when symptoms are refractory, fertility is a priority, imaging becomes suspicious, or definitive diagnosis and treatment are required [139]. Second, persistent symptoms may reflect recurrence, residual disease, or ongoing biological activity despite treatment [140]. Third, pain may be driven predominantly by peripheral or central sensitization, pelvic floor dysfunction, visceral hypersensitivity, or overlapping chronic pain disorders, which may not respond sufficiently to hormonal suppression alone [141].

This distinction is clinically important. Patients with poor response should undergo careful reassessment rather than automatic escalation to repeated surgery. Reassessment may include review of adherence and treatment duration, repeat expert ultrasound, MRI when indicated, evaluation for adenomyosis or deep disease, assessment of pelvic floor dysfunction, gastrointestinal or urinary evaluation, and consideration of multidisciplinary pain management.

The ongoing uncertainty regarding the benefit of surgery for superficial peritoneal disease, particularly in pain-dominant presentations, further supports a selective approach. Current research continues to investigate whether laparoscopic excision of superficial peritoneal lesions provides meaningful benefit beyond placebo or nonspecific surgical effects in selected patients [142]. Until stronger phenotype-specific evidence is available, surgical decisions should be individualized.

### 6.5. Role in an Integrated Decision Pathway

Response to empirical hormonal therapy may be useful within an integrated decision pathway for suspected SPE, but only as one element among several. It should not be interpreted as a stand-alone diagnostic marker. Instead, it may help stratify patients according to clinical evolution and guide subsequent management.

Patients with good symptom control, no infertility, no suspicious imaging findings, and no red flags may continue conservative medical management with follow-up. In these cases, avoiding immediate diagnostic laparoscopy may be appropriate after shared decision-making [139,140].

Patients with partial response, recurrent symptoms, infertility, persistent functional impairment, or discordant clinical and imaging findings may require referral to an endometriosis reference center, repeat expert imaging, MRI, or selective laparoscopy. Patients with diffuse pain, poor correlation between symptoms and imaging, pelvic floor tenderness, visceral hypersensitivity, or features suggestive of central sensitization may benefit from multidisciplinary pain assessment rather than surgery alone [140,141,142].

Therefore, medical therapy response should be framed as a low-specificity functional signal. It may contribute to patient stratification, but it cannot define biologically active SPE by itself. Its greatest value lies in helping clinicians distinguish patients who may be managed conservatively from those requiring further imaging, specialized referral, multidisciplinary pain care, or selective surgical exploration [143,144,145,146].

## 7. Liquid Biopsy, Molecular Biomarkers, and Microbiome: Promise, Heterogeneity, and Current Limitations

### 7.1. Liquid Biopsy and Circulating Biomarkers

The development of non-invasive biomarkers for endometriosis has attracted increasing interest, particularly in patients with suspected superficial peritoneal endometriosis, in whom conventional imaging may be negative and diagnostic confirmation often requires laparoscopy [147,148,149,150]. In this setting, molecular approaches have been investigated as potential adjunctive tools to improve patient stratification, reduce diagnostic delay, and better characterize biological disease heterogeneity [151,152,153,154,155].

Liquid biopsy approaches include the analysis of circulating microRNAs, extracellular vesicles, exosomes, cell-free DNA, inflammatory mediators, proteomic signatures, metabolomic profiles, adipokine-related markers, and epigenetic signals obtained from peripheral blood or other biological fluids [148,149,150,151,152]. In theory, these biomarkers may help identify inflammatory, neuroangiogenic, immune, hormonal, or metabolic patterns associated with endometriosis-related disease activity.

This perspective is particularly attractive in SPE because superficial lesions are frequently small, multifocal, and poorly visible using conventional imaging. A molecular signal, if validated, could potentially provide information on biological activity even when lesion visibility is limited. However, this remains a research hypothesis rather than a current clinical tool.

At present, no single circulating biomarker has demonstrated sufficient sensitivity, specificity, reproducibility, and external validation to replace diagnostic laparoscopy or to establish a definitive diagnosis of SPE. Most available studies are limited by small sample size, heterogeneous populations, variable definitions of endometriosis phenotypes, differences in disease stage, lack of standardized sampling protocols, and inconsistent adjustment for relevant clinical confounders.

Therefore, liquid biopsy should currently be interpreted as an investigational approach. Its potential clinical role is not to confirm SPE independently, but to support future phenotype-specific risk stratification after adequate validation.

### 7.2. Phenotype-Dependent Interpretation of Biomarkers

A major limitation of biomarker research in endometriosis is disease heterogeneity. Ovarian endometriosis, deep infiltrating endometriosis, and superficial peritoneal endometriosis differ substantially in lesion morphology, fibrosis, vascularization, inflammatory activity, immune microenvironment, hormonal responsiveness, and molecular profile. Consequently, biomarkers derived from mixed endometriosis populations may not be directly applicable to suspected SPE.

Future biomarker studies should therefore move beyond the search for universal diagnostic markers and consider phenotype-dependent interpretation. Biomarker levels may be influenced by pain phenotype, infertility status, lesion type, disease stage, inflammatory activity, menstrual cycle phase, hormonal treatment exposure, previous surgery, BMI, and comorbid conditions [150].

Infertility represents a particularly important confounder. Some biomarkers may reflect altered peritoneal inflammation, oocyte microenvironment, metabolic state, or immune activation related to infertility rather than SPE itself. Similarly, pain-dominant and infertility-dominant phenotypes may show different biological signatures. For this reason, biomarker studies should stratify patients according to clinical presentation rather than grouping all endometriosis phenotypes together.

Recent data also suggest that adipokine-related markers may show phenotype-dependent associations. For example, the leptin/BMI ratio measured in plasma and peritoneal fluid has been investigated in relation to endometriosis and infertility. These findings suggest that metabolic and inflammatory markers may vary according to clinical context and biological compartment, while their diagnostic utility as stand-alone biomarkers remains limited and requires further validation [151].

BMI is another relevant variable. Adipose tissue influences systemic inflammation, leptin signaling, estrogen metabolism, insulin resistance, and immune function. Consequently, circulating inflammatory or adipokine-related markers may be significantly affected by body composition. Failure to adjust for BMI may reduce reproducibility and contribute to conflicting biomarker results across studies.

### 7.3. Menstrual Cycle, Hormonal Treatment, and Biological Sample Type

Menstrual cycle phase represents a critical methodological issue in biomarker research. Endometriosis is an estrogen-dependent and hormonally dynamic disease; therefore, circulating inflammatory mediators, microRNAs, extracellular vesicles, immune markers, and epigenetic signals may vary across the follicular, periovulatory, and luteal phases. Without standardized timing of sample collection, biomarker results may be difficult to interpret and compare [133].

Hormonal treatment exposure is another major confounder. Combined oral contraceptives, progestins, dienogest, levonorgestrel-releasing intrauterine systems, GnRH agonists, and GnRH antagonists may modify inflammatory activity, ovarian function, bleeding patterns, endometrial biology, immune signaling, and circulating molecular profiles. Therefore, biomarker studies should clearly report current and previous hormonal exposure, treatment washout periods, and timing of sample collection relative to therapy [134].

The type of biological material analyzed also strongly influences biomarker interpretation [155]. Serum, plasma, whole blood, urine, menstrual effluent, peritoneal fluid, extracellular vesicles, exosomes, endometrial tissue, and lesion tissue may provide different biological information. Peritoneal fluid may better reflect the local inflammatory microenvironment, whereas plasma or serum may be more clinically accessible but less specific. Menstrual effluent may capture endometrial and immune signals, but collection and standardization remain challenging [156]. Extracellular vesicle and exosomal markers may offer more specific molecular information, but isolation methods and analytical platforms remain heterogeneous.

These methodological differences are particularly relevant for SPE. A biomarker detectable in peritoneal fluid may not be easily translated into routine clinical practice, whereas a circulating marker may be clinically feasible but insufficiently specific for superficial lesions. Future studies should therefore clearly define the biological source, collection protocol, processing method, analytical platform, and intended clinical use of each biomarker [157].

### 7.4. Microbiome and SPE-Related Biological Activity

The microbiome has emerged as another potential contributor to endometriosis pathophysiology, through interactions among immune activation, estrogen metabolism, epithelial barrier function, inflammatory signaling, and local pelvic microenvironment [153,154]. Alterations in gut, reproductive tract, and peritoneal microbiota have been described in endometriosis, suggesting that microbial communities may modulate inflammatory and hormonal pathways relevant to disease persistence and symptom generation [152,153,154,155,156].

In this context, Fusobacterium has recently attracted attention as a potential microbial factor involved in endometriosis development. Muraoka et al. reported that Fusobacterium infection may facilitate the development of endometriosis through phenotypic transition of endometrial fibroblasts, suggesting a possible mechanistic link between microbial exposure, tissue remodeling, and lesion formation [158]. This observation is biologically intriguing and supports the broader concept that microbial–immune interactions may contribute to endometriosis pathogenesis.

However, the relevance of Fusobacterium and other microbiome-related signatures to SPE specifically remains uncertain. Most microbiome studies include mixed endometriosis phenotypes or do not provide separate analyses for superficial, ovarian, and deep disease. In addition, microbiome profiles may be influenced by antibiotics, diet, bowel symptoms, sexual activity, hormonal treatment, menstrual cycle phase, sample site, and laboratory methods.

Therefore, microbiome findings should be interpreted as experimental and hypothesis-generating. They may help identify future biological pathways involved in inflammation and lesion persistence, but they are not currently suitable for clinical diagnosis, phenotyping, or treatment selection in suspected SPE.

### 7.5. From Single Biomarkers to Integrated Panels

Given the complexity of SPE, multimarker panels are likely to be more informative than isolated biomarkers. A clinically useful panel would ideally combine markers reflecting different biological domains, including inflammation, neuroangiogenesis, hormonal responsiveness, immune dysregulation, oxidative stress, epigenetic regulation, microbiome-related signals, metabolic status, and pain sensitization [157,158,159].

However, even biomarker panels require rigorous validation. They should be tested prospectively in well-characterized cohorts, with separate analysis of superficial, ovarian, and deep phenotypes. Diagnostic performance should be reported using standardized measures, including sensitivity, specificity, positive predictive value, negative predictive value, calibration, reproducibility, and external validation [150,151,152,153].

Importantly, panels should be evaluated not only for their ability to detect endometriosis in general, but also for their capacity to improve clinical decision-making in specific scenarios, such as suspected SPE with negative imaging, persistent symptoms despite empirical treatment, infertility, or discordance between symptom severity and anatomical findings.

In this context, biomarkers should not be viewed as stand-alone diagnostic substitutes for laparoscopy. Their future value may lie in risk stratification, identification of biological phenotypes, prediction of treatment response, monitoring of disease activity, or selection of patients who may benefit from surgery, medical therapy, or multidisciplinary pain management [148,149].

### 7.6. Multi-Omics, Artificial Intelligence, and Future Molecular Stratification

Because no single biomarker is likely to capture the biological complexity of endometriosis, current research is progressively shifting toward multi-omics and computational approaches [160,161]. Multi-omics strategies integrate genomic, epigenomic, transcriptomic, proteomic, metabolomic, microbiomic, and immunologic information to identify broader biological signatures of disease activity [162,163,164].

In suspected SPE, such approaches may be particularly useful because clinically relevant disease may be difficult to visualize anatomically. A systems-based model could theoretically integrate molecular data with symptom phenotype, dynamic ultrasound findings, pain distribution, hormonal treatment response, infertility status, and clinical history. However, this remains a future objective rather than an available clinical standard.

Artificial intelligence and machine learning may eventually help analyze these multidimensional datasets and identify patterns that are not apparent using conventional statistical methods [165,166,167]. Potential applications include prediction of disease probability, identification of biological phenotypes, estimation of treatment response, recurrence risk stratification, and selection of patients more likely to benefit from surgical versus conservative management.

Nevertheless, these technologies remain investigational. Their implementation in SPE is limited by small and heterogeneous datasets, inconsistent diagnostic ground truth, lack of phenotype-specific validation, risk of algorithmic bias, and uncertain clinical utility. Before integration into clinical practice, AI-based models must demonstrate reproducibility, transparency, external validation, and added value beyond expert clinical assessment, imaging, and shared decision-making [168].

### 7.7. Clinical Readiness and Future Directions

At present, liquid biopsy, molecular biomarkers, microbiome profiling, multi-omics, and AI-based models remain investigational in suspected SPE. Their incorporation into routine clinical practice is limited by insufficient validation, lack of standardized thresholds, heterogeneity of patient populations, variability in biological samples, and uncertain incremental value over expert clinical assessment and imaging [123,140,155].

The role of these tools should therefore be described cautiously. They represent promising research directions and may eventually contribute to integrated functional and molecular stratification, but they are not currently ready to guide routine diagnostic or therapeutic decisions in SPE.

Future studies should prioritize prospective, phenotype-specific designs; standardized sample collection across menstrual cycle phases; careful adjustment for BMI, infertility, hormonal treatment, previous surgery, and comorbid pain disorders; comparison between biological compartments; and validation of multimarker panels in independent cohorts [151,152,153,154]. Only after such validation could molecular approaches become clinically meaningful components of a personalized diagnostic pathway for suspected superficial peritoneal endometriosis.

## 8. Discussion: Clinical Implications and Future Research Agenda

### 8.1. From Anatomical Diagnosis to Cautious Multidimensional Interpretation

The evolving understanding of superficial peritoneal endometriosis is challenging the traditional assumption that disease activity can be fully interpreted through direct visualization of lesions alone. Although anatomical identification of endometriotic implants remains essential, visible lesions may provide only a partial representation of disease activity, clinical impact, and long-term behavior [66,67,68,69].

Historically, endometriosis has largely been interpreted through a lesion-based surgical paradigm, in which disease severity was considered mainly dependent on the extent and distribution of visible implants. Within this framework, laparoscopy became the diagnostic reference standard because it allowed direct visualization and surgical confirmation of peritoneal lesions [51]. However, this structural model does not fully explain several common clinical scenarios, including severe pain in women with minimal superficial disease, persistent symptoms after apparently complete surgical treatment, negative imaging despite strong symptoms, and heterogeneous responses to hormonal therapy among patients with similar anatomical findings [70,71,72,73,74].

These observations suggest that symptom generation in superficial endometriosis cannot be explained by lesion burden alone. Chronic inflammation, neuroangiogenesis, immune dysregulation, hormonal imbalance, epigenetic alterations, peripheral sensitization, and central nervous system modulation may contribute to pain persistence and disease heterogeneity [100,101,102,103,104,105]. Nevertheless, these mechanisms should not be interpreted as diagnostic proof of SPE. They provide a biological rationale for symptom heterogeneity, but they remain insufficient to define a validated clinical diagnostic category.

Therefore, the diagnostic focus should not shift away from laparoscopy, but rather toward a more cautious multidimensional interpretation of suspected SPE. Anatomical findings should be interpreted together with symptom phenotype, expert imaging, exclusion of alternative pain generators, response to empirical treatment, fertility goals, and, in the future, validated molecular tools.

### 8.2. Integration of Clinical Phenotype, Imaging, Treatment Response, and Molecular Research

Within this cautious framework, clinical symptom phenotype remains fundamental. Dysmenorrhea, chronic pelvic pain, deep dyspareunia, cyclic gastrointestinal symptoms, acyclic pelvic discomfort, fatigue, and quality-of-life impairment may all provide relevant clinical information [76]. However, symptom severity alone cannot establish the diagnosis of SPE, because chronic pelvic pain may arise from multiple gynecological, gastrointestinal, urinary, musculoskeletal, neurological, and centralized pain mechanisms [1,2,3].

Functional imaging may provide useful adjunctive information. Dynamic transvaginal ultrasound, sliding sign evaluation, tenderness-guided examination, compartment mobility assessment, and functional soft markers may identify indirect manifestations of pelvic adhesions, restricted mobility, or site-specific pain even in the absence of overt endometriomas or deep infiltrating lesions [23,27,33]. However, these findings remain operator-dependent and incompletely standardized for SPE. They should therefore be used to support clinical reasoning and referral decisions, not as stand-alone diagnostic criteria.

Therapeutic response may also contribute to clinical interpretation, but only as a low-specificity functional signal [8]. Improvement under hormonal suppression may suggest hormonally modulated pelvic pain or estrogen-dependent inflammatory activity, whereas persistent symptoms despite adequate therapy may reflect central sensitization, pelvic floor dysfunction, visceral hypersensitivity, treatment resistance, occult surgically relevant disease, or mixed pain mechanisms [83,84,85,86]. Consequently, response to medical therapy may support individualized decision-making, but it should not be considered diagnostic confirmation of biologically active SPE.

Emerging molecular approaches may further refine this process in the future. Circulating inflammatory signatures, epigenetic alterations, microRNA profiles, extracellular vesicles, microbiome analysis, and multi-omics models may eventually help characterize biological phenotypes beyond lesion morphology [150,151,152,153,154,155]. At present, however, these tools remain investigational and should not be used as substitutes for laparoscopy or established clinical evaluation.

### 8.3. Limitations of Current Evidence

Despite growing interest in functional and molecular approaches to superficial endometriosis, several important limitations must be acknowledged. First, SPE remains intrinsically difficult to study because lesions are frequently subtle, heterogeneous, multifocal, and inconsistently classified [23,24,25]. This complexity contributes to substantial variability across studies investigating imaging findings, inflammatory pathways, biomarkers, and molecular signatures. Many currently available studies include relatively small cohorts, mixed endometriosis phenotypes, and heterogeneous diagnostic criteria, thereby limiting generalizability.

Second, although molecular biomarkers and liquid biopsy approaches represent promising research areas, most proposed markers currently lack sufficient external validation, reproducibility, and diagnostic performance for routine clinical implementation [150]. Sensitivity and specificity remain variable, and many molecular alterations identified in endometriosis overlap with those observed in other inflammatory, gynecological, metabolic, or chronic pain conditions. This overlap substantially limits diagnostic specificity, particularly for SPE [27,28,29,30,31,32].

Similarly, dynamic ultrasound assessment and interpretation of functional pelvic soft markers remain highly operator-dependent. Standardized protocols for evaluating superficial disease are still evolving, and interobserver variability continues to represent an important limitation [41,42,43,44,45]. Sliding sign assessment, pelvic organ mobility evaluation, tenderness-guided ultrasound, and functional soft markers may provide useful indirect information, but they should not be interpreted as validated diagnostic criteria for SPE [33,48].

The concept of biologically active occult disease also requires careful interpretation in order to avoid indiscriminate overdiagnosis [149,150,151,152]. Chronic pelvic pain is inherently multifactorial, and several overlapping disorders may coexist in women with suspected endometriosis, including adenomyosis, pelvic floor dysfunction, irritable bowel syndrome, bladder pain syndrome, vulvodynia, fibromyalgia, and central sensitization syndromes [46,47,48,49]. For this reason, careful multidisciplinary evaluation remains essential, particularly in patients with negative imaging, diffuse symptoms, poor response to hormonal therapy, or discordance between pain severity and anatomical findings [8,9,10,11].

Finally, although reducing unnecessary surgery represents an important objective, the role of laparoscopy should not be underestimated. Surgical evaluation remains fundamental in carefully selected patients, particularly when infertility, severe refractory symptoms, organ involvement, uncertain diagnosis, suspicious imaging findings, or suspicion of complex disease are present. Therefore, current evidence supports a more selective use of laparoscopy rather than its replacement by functional or molecular tools.

### 8.4. Toward a Cautious Integrated Framework

Taken together, the evidence reviewed in this article supports a cautious transition toward a more integrated interpretation of superficial peritoneal endometriosis. Rather than representing a purely anatomical condition identifiable only through surgical visualization, SPE may involve complex interactions among visible lesions, local inflammation, immune dysfunction, neuroangiogenesis, hormonal signaling, epigenetic regulation, peripheral sensitization, and central pain-processing mechanisms [63,64,65,150].

Within this framework, visible lesions remain clinically important, but they may represent only one component of a broader biological and functional process. In selected patients, symptoms may be influenced by inflammatory signaling, neuroimmune activation, sensitization mechanisms, and hormonal responsiveness, even when anatomical abnormalities are minimal or difficult to detect. However, this concept remains hypothesis-generating and should not be interpreted as a validated diagnostic model [166,167,168].

This evolving perspective has potential clinical implications because it encourages the integration of clinical phenotype, expert imaging, empirical treatment response, pain assessment, and future molecular research into individualized diagnostic pathways [107,108,109,110,111]. At present, however, these components have different levels of evidence. Laparoscopy remains the reference standard for direct visualization of superficial peritoneal lesions. Dynamic ultrasound and treatment response may serve as adjunctive functional observations. Molecular biomarkers, microbiome profiling, multi-omics approaches, and artificial intelligence remain investigational [163,164,165].

Most importantly, this framework challenges the assumption that every symptomatic patient with suspected SPE and negative imaging necessarily requires immediate surgical confirmation before meaningful management can begin [66]. In selected patients without infertility, suspicious imaging findings, red flags, or refractory symptoms, empirical medical treatment and careful follow-up may be appropriate after shared decision-making. Conversely, persistent symptoms, infertility, organ-related symptoms, imaging abnormalities, or failure of conservative management may justify referral to an endometriosis reference center, MRI, multidisciplinary evaluation, or selective laparoscopy [18].

Ultimately, the central challenge is not only to visualize superficial lesions, but to identify which patients are most likely to benefit from surgery and which may be better served by conservative, medical, imaging-based, or multidisciplinary pain-oriented strategies. Future validated models may help integrate anatomical, functional, clinical, and molecular information, but such models are not yet ready to replace current diagnostic standards (Table 3).

## 9. Conclusions

Superficial peritoneal endometriosis remains one of the most challenging endometriosis phenotypes to diagnose and manage because lesions are frequently subtle, multifocal, biologically heterogeneous, and poorly detectable using conventional imaging. Diagnostic laparoscopy therefore continues to represent the reference standard for the direct visualization and treatment of superficial peritoneal lesions in appropriately selected patients.

At the same time, a purely lesion-based model may be incomplete when used alone to interpret symptom severity, pain persistence, and clinical heterogeneity. In suspected or confirmed SPE, symptoms may reflect a complex interaction between visible lesions, local inflammation, neuroangiogenesis, immune dysregulation, hormonal responsiveness, peripheral sensitization, central sensitization, and overlapping pain disorders. This complexity is particularly relevant in patients with chronic pelvic pain and negative imaging findings, in whom symptoms should not automatically be attributed to occult superficial disease.

Dynamic transvaginal ultrasound, sliding sign assessment, pelvic organ mobility evaluation, tenderness-guided examination, and functional soft markers may provide indirect information regarding pelvic mobility, adhesions, compartmental restriction, and site-specific pain. However, these approaches remain operator-dependent and insufficiently standardized for SPE. They should therefore be interpreted as adjunctive functional observations rather than validated diagnostic tools.

Similarly, response to empirical hormonal therapy may support clinical reasoning in selected patients, but it has low specificity and should not be considered diagnostic confirmation of SPE. Symptom improvement may also reflect improvement of adenomyosis, primary dysmenorrhea, ovulation-related pain, abnormal uterine bleeding, or other estrogen-sensitive conditions.

Molecular biomarkers, liquid biopsy, microbiome profiling, multi-omics approaches, and artificial intelligence represent promising research areas. Nevertheless, they remain investigational and are not currently ready to guide routine diagnosis or treatment decisions in suspected SPE. Their future clinical translation will require phenotype-specific validation, standardized sampling, adjustment for relevant confounders, and demonstration of added value over expert clinical assessment and imaging.

The future of SPE management should therefore not be framed as the replacement of laparoscopy by functional or molecular tools. Rather, the goal should be to develop validated, integrated models that improve patient selection. Such models may eventually combine symptom phenotype, expert imaging, empirical treatment response, fertility goals, exclusion of alternative pain generators, patient preferences, and validated biomarkers.

Until such models are available, suspected SPE should be approached through cautious individualized assessment. Laparoscopy remains essential when definitive diagnosis or surgical treatment is required, particularly in patients with infertility, refractory symptoms, suspicious imaging findings, suspected complex disease, or failure of conservative management. Conversely, conservative medical management, expert follow-up, MRI, referral to an endometriosis reference center, or multidisciplinary pain care may be appropriate in selected scenarios. This balanced strategy may help reduce unnecessary procedures while preserving timely diagnosis and appropriate treatment for patients most likely to benefit from surgery.

## Figures and Tables

**Table 1:** Indirect Functional Ultrasound Findings in Suspected Superficial Peritoneal Endometriosis: Potential Clinical Meaning and Limitations.

Functional Marker	Ultrasound Finding	Potential Biological Meaning	Clinical Implication	Main Limitation
Negative sliding sign [27]	Reduced or absent gliding between pelvic organs	Adhesions, chronic inflammation, occult peritoneal involvement	May increase clinical suspicion of impaired pelvic mobility, but remains a non-specific indirect finding requiring clinical correlation	Low specificity in patients with prior surgery or non-endometriotic adhesions
Ovarian fixation [33]	Reduced ovarian mobility or adherence to pelvic sidewall/uterus	Inflammatory adhesions involving ovarian fossa or posterior compartment	Non-specific indirect sign of reduced ovarian mobility, even without endometrioma; requires clinical correlation.	Operator-dependent assessment
Site-specific tenderness [23]	Reproduction of pain during targeted probe pressure	Peripheral sensitization and local neuroinflammatory activation	Correlates anatomical areas with symptom generation	Subjective patient response
Compartment stiffness [16]	Reduced flexibility of posterior or lateral pelvic compartments	Fibrosis, chronic inflammation, altered tissue biomechanics	May reflect persistent inflammatory activity	Difficult to standardize quantitatively
Altered uterine mobility [17]	Reduced mobility during dynamic examination	Pelvic adhesions or chronic inflammatory remodeling	Suggests functional pelvic impairment	Can overlap with adenomyosis or prior surgery
Posterior compartment tenderness [32]	Pain elicited in pouch of Douglas or uterosacral region	Occult inflammatory or neuroangiogenic disease	Non-specific indirect finding that may indicate posterior compartment tenderness; requires clinical correlation.	Limited specificity
Asymmetric organ movement [25]	Non-physiological movement of pelvic structures during dynamic assessment	Localized adhesions or compartmental dysfunction	Supports functional pelvic abnormality	Limited reproducibility
Mild ovarian medialization [22]	Ovaries positioned closer to uterus without classic kissing ovaries	Early adhesive process or subtle posterior inflammation	Non-specific indirect sign of altered pelvic organ position; requires clinical correlation.	Often subtle and non-specific
Reduced bowel sliding [38]	Limited rectosigmoid mobility despite absence of DIE nodules	Minimal inflammatory adhesions or early posterior disease	May precede overt deep infiltrating disease	Difficult interpretation in functional bowel disorders
Pain-guided ultrasound positivity [28]	Concordance between dynamic examination and symptom reproduction	Functional neuroinflammatory pelvic activation	Strengthens clinical suspicion despite negative morphology	Requires experienced operator and standardized approach

DIE, deep infiltrating endometriosis.

**Table 2:** Molecular and Neuroinflammatory Mechanisms Underlying Pain in Superficial Peritoneal Endometriosis.

Mechanism	Main Mediators/Pathways	Biological Effect	Clinical Consequence	Potential Therapeutic Implication
Chronic inflammation [108]	IL-1β, IL-6, TNF-α, prostaglandins, NF-κB	Persistent inflammatory activation and cytokine production	Dysmenorrhea, chronic pelvic pain, inflammatory pain amplification	Hormonal suppression, anti-inflammatory therapies
Neuroangiogenesis [94,95,96,97]	VEGF, NGF, BDNF	Growth of sensory nerve fibers and vascular proliferation	Increased nociceptive signaling and pain sensitivity	Anti-neuroangiogenic and anti-inflammatory approaches
Peripheral sensitization [98]	Cytokines, prostaglandins, ion channel modulation	Lowered activation threshold of nociceptive fibers	Hyperalgesia and exaggerated pain responses	Neuromodulatory and anti-inflammatory strategies
Central sensitization [99,100]	Spinal cord hyperexcitability, altered central pain processing	Persistent amplification of pain independent of lesion burden	Chronic pelvic pain persistence after surgery or therapy	Multidisciplinary chronic pain management
Mast cell activation [114]	Histamine, tryptase, prostaglandins, NGF	Neuroimmune interaction and neurogenic inflammation	Severe tenderness and pain amplification	Mast cell modulation (investigational)
Macrophage dysregulation [109]	M2 macrophages, VEGF, cytokines	Chronic inflammation, angiogenesis, lesion support	Persistent inflammatory microenvironment	Immune-targeted therapeutic approaches
Oxidative stress [103]	Reactive oxygen species (ROS), mitochondrial dysfunction	Cellular injury and inflammatory amplification	Pain chronification and tissue remodeling	Antioxidant and anti-inflammatory strategies
Estrogen-dependent signaling [116]	ERβ overexpression, aromatase activation	Enhanced inflammatory and neuroangiogenic stimulation	Hormone-dependent pain activity	Progestins, GnRH agonists/antagonists
Progesterone resistance [117]	Reduced PR expression, altered progesterone signaling	Failure of anti-inflammatory hormonal regulation	Persistent inflammatory activity and treatment resistance	Personalized hormonal strategies
Epigenetic reprogramming [118,119]	DNA methylation, histone modification, miRNAs	Stable maintenance of inflammatory phenotype (“molecular memory”)	Persistent biologically active disease despite minimal lesions	Future epigenetic-targeted therapies
Immune dysfunction [105]	Reduced NK activity, Treg expansion	Impaired clearance of ectopic endometrial cells	Persistence of occult inflammatory disease	Immunomodulatory approaches
Neuroimmune crosstalk [121]	Cytokine–nerve interaction, mast cell–nerve signaling	Self-sustaining inflammatory pain loops	Chronic pelvic pain maintenance	Combined anti-inflammatory and neuromodulatory treatment

BDNF, brain-derived neurotrophic factor; ERβ, estrogen receptor beta; IL, interleukin; NF-κB, nuclear factor kappa B; NGF, nerve growth factor; NK, natural killer; PR, progesterone receptor; ROS, reactive oxygen species; TNF-α, tumor necrosis factor alpha; Treg, regulatory T cell; VEGF, vascular endothelial growth factor.

**Table 3:** Established, adjunctive, and investigational components in the assessment of suspected superficial peritoneal endometriosis.

Component	Current Evidence Status	Potential Clinical Contribution	Main Limitations/Comments
Disease definition [48]	SPE is currently defined primarily by the anatomical presence of superficial peritoneal lesions, usually identified at surgery.	Future interpretation may incorporate biological, inflammatory, neuroimmune, and functional dimensions.	The concept of “biologically active SPE” remains provisional and should not be considered a validated clinical diagnostic category.
Diagnostic laparoscopy [51]	Established clinical reference standard for direct visualization of superficial peritoneal lesions.	Provides anatomical assessment, histological confirmation when biopsies are performed, and simultaneous treatment in selected patients.	It is invasive and should be used selectively; visible lesions do not always fully explain symptoms.
Main diagnostic target [12,13,14,42,43,44,45,46,47,48]	Current diagnosis remains mainly focused on lesion visualization and surgical confirmation.	Future models may aim to identify clinically meaningful disease activity and improve patient selection.	Functional and molecular tools are not yet validated substitutes for direct surgical visualization.
Interpretation of pain [23,28]	Pain has traditionally been interpreted mainly in relation to lesion burden.	A broader interpretation may integrate inflammation, neuroangiogenesis, sensitization, and molecular dysregulation.	Pain is multifactorial and cannot be attributed to SPE alone without considering overlapping pain disorders.
Role of imaging [32,33,34,35,36]	Conventional imaging is effective for ovarian endometriosis and DIE, but limited for direct visualization of superficial lesions.	Functional pelvic assessment may provide indirect information regarding adhesions, mobility restriction, and site-specific pain.	Indirect findings are not diagnostic of SPE and remain incompletely standardized.
Dynamic ultrasound, sliding sign, mobility, and tenderness-guided evaluation [23,48,66]	Adjunctive but incompletely validated in suspected SPE.	May support clinical reasoning, referral to expert centers, MRI indication, or selective laparoscopy.	Operator-dependent; sensitivity, specificity, reproducibility, and clinical thresholds for SPE remain insufficiently defined.
Hormonal therapy response [133,134,135,136,137]	Established as symptomatic treatment in endometriosis-associated pain.	May provide a low-specificity functional signal suggesting hormonally modulated pelvic pain.	Response is not specific for SPE and may also reflect adenomyosis, primary dysmenorrhea, anti-ovulatory effects, or reduced bleeding.
Partial or absent response to therapy [101,102,103]	Clinically relevant but non-specific.	May prompt reassessment, repeat expert imaging, MRI, referral, multidisciplinary evaluation, or selective surgery.	Does not exclude endometriosis; may reflect treatment resistance, central sensitization, overlapping pain disorders, or surgically relevant disease.
Pathophysiological focus [107,108,109,110]	Traditional models focused mainly on visible ectopic implants.	Current evidence supports interest in inflammation, neuroimmune activation, progesterone resistance, and epigenetic regulation.	These mechanisms improve biological understanding but are not yet clinically actionable diagnostic criteria for SPE.
Neuroinflammation and sensitization [99,100,101]	Biologically plausible and increasingly supported in endometriosis-associated pain.	May help explain pain persistence and discordance between symptom severity and lesion burden.	Not specific to SPE and should not be used alone to infer occult superficial disease.
Persistence or recurrence of symptoms [140,141]	Traditionally interpreted mainly as residual or recurrent lesions.	May also reflect persistent inflammatory, neuroimmune, or sensitization mechanisms.	Surgery alone may not fully reverse symptoms in all patients.
Molecular biomarkers and liquid biopsy [150]	Investigational.	Potential future role in non-invasive diagnosis, biological phenotyping, and patient stratification.	Insufficient external validation; interpretation is limited by phenotype heterogeneity, BMI, menstrual cycle, infertility, hormonal exposure, disease stage, and sample type.
Microbiome and epigenetics [119,125]	Investigational.	May offer translational insights into inflammatory, immune, hormonal, and microbial–host interactions.	Current relevance to SPE specifically remains uncertain; phenotype-specific validation is lacking.
Surgical objective [51]	Confirmation and treatment of visible lesions.	May become more selective within individualized management pathways.	Surgery remains essential in selected patients, especially with infertility, refractory symptoms, suspicious imaging, or complex disease.
Therapeutic strategy [135]	Traditionally based on symptom control, hormonal suppression, and surgery when indicated.	Future management may become increasingly phenotype-oriented and multidisciplinary.	Precision-based approaches remain a future goal rather than a current standard.
Overall clinical framework [136]	Current practice remains primarily based on anatomical diagnosis, clinical assessment, imaging, and selective surgery.	A future integrated framework may combine anatomical, functional, clinical, and molecular information.	This integrated model remains conceptual and should not be interpreted as a validated replacement for current diagnostic standards.

SPE, superficial peritoneal endometriosis; DIE, deep infiltrating endometriosis; MRI, magnetic resonance imaging.

## Data Availability

The present review was based on published articles. All summary data generated during this study are included in this published article. The data discussed in this narrative review are available in the original articles cited.

## Abbreviations

BDNF: Brain-Derived Neurotrophic Factor; COX-2, Cyclooxygenase-2; CPP, Chronic Pelvic Pain; DIE, Deep Infiltrating Endometriosis; ERβ, Estrogen Receptor Beta; GnRH, Gonadotropin-Releasing Hormone; IL, Interleukin; MRI, Magnetic Resonance Imaging; NF-κB, Nuclear Factor Kappa B; NGF, Nerve Growth Factor; NK, Natural Killer; PR, Progesterone Receptor; ROS, Reactive Oxygen Species; SPE, Superficial Peritoneal Endometriosis; TNF-α, Tumor Necrosis Factor Alpha; Treg, Regulatory T Cell; TVUS, Transvaginal Ultrasound; VEGF, Vascular Endothelial Growth Factor.
